# Long-term safety and tolerability of bapineuzumab in patients with Alzheimer’s disease in two phase 3 extension studies

**DOI:** 10.1186/s13195-016-0193-y

**Published:** 2016-06-23

**Authors:** Adrian Ivanoiu, Jérémie Pariente, Kevin Booth, Kasia Lobello, Gerald Luscan, Lisa Hua, Prisca Lucas, Scot Styren, Lingfeng Yang, David Li, Ronald S. Black, H. Robert Brashear, Thomas McRae

**Affiliations:** Université Catholique de Louvain, Cliniques Universitaires Saint-Luc, 10 Avenue Hipocrate, B-1200 Bruxelles, Belgium; Centre d’investigation Clinique, Centre Mémoire et Langage, Service de Neurologie, CHU Purpan, Place du Dr Baylac, 31059 Toulouse, France; Pfizer Inc., 500 Arcola Road, Collegeville, PA 19426 USA; Pfizer PGRD, 23-25 avenue du Docteur Lannelongue, 75668 Paris cedex 14, France; Pfizer Inc., Eastern Point Road, Groton, CT 06340 USA; Janssen Alzheimer Immunotherapy Research & Development, LLC, 700 Gateway Blvd., South San Francisco, CA 94080 USA; Pfizer Inc., 235 East 42nd Street, New York, NY 10017 USA

**Keywords:** Alzheimer’s disease, Amyloid beta, Amyloid-related imaging abnormalities with edema or effusions/vasogenic edema, Bapineuzumab, Immunotherapy

## Abstract

**Background:**

Immunotherapy with monoclonal antibodies that target amyloid beta has been under investigation as a treatment for patients with Alzheimer’s disease (AD). The 3000 and 3001 phase 3 clinical studies of intravenous bapineuzumab assessed safety and efficacy in patients with mild to moderate AD recruited in over 26 countries. This article describes the long-term safety and tolerability of bapineuzumab in the extension studies for these two protocols.

**Methods:**

The long-term safety and tolerability of intravenous-administered bapineuzumab in patients with AD was evaluated in apolipoprotein E ε4 allele noncarriers (Study 3002, extension of Study 3000) and apolipoprotein E ε4 allele carriers (Study 3003, extension of Study 3001). Those receiving bapineuzumab in the parent study were continued at the same dose; if receiving placebo, patients began bapineuzumab. Bapineuzumab doses were 0.5 mg/kg in both studies and also 1.0 mg/kg in the noncarrier study. Clinical efficacy of bapineuzumab was also assessed in exploratory analyses.

**Results:**

Because of lack of efficacy in two other phase 3 trials, the parent protocols were stopped early. As a result, Studies 3002 and 3003 were also terminated. In total, 492 and 202 patients were enrolled in Studies 3003 and 3002, respectively. In apolipoprotein E ε4 carriers (Study 3003), treatment-emergent adverse events occurred in 70.7 % of the patients who originally received placebo and 66.9 % of those who originally received bapineuzumab. In noncarriers, treatment-emergent adverse events occurred in 82.1 % and 67.6 % of patients who received placebo + bapineuzumab 0.5 mg/kg and placebo + bapineuzumab 1.0 mg/kg, respectively, and in 72.7 % and 64.3 % of those who received bapineuzumab + bapineuzumab 0.5 mg/kg and 1.0 mg/kg, respectively. Amyloid-related imaging abnormalities with edema or effusions were the main bapineuzumab-associated adverse events in both studies, occurring in approximately 11 % of placebo + bapineuzumab and 4 % of bapineuzumab + bapineuzumab groups overall. Exploratory analyses of clinical efficacy were not significantly different between groups in either study.

**Conclusions:**

In these phase 3 extension studies, intravenous bapineuzumab administered for up to approximately 3 years showed no unexpected safety signals and a safety profile consistent with previous bapineuzumab trials.

**Trial registration:**

Noncarriers (Study 3002): ClinicalTrials.gov NCT00996918. Registered 14 October 2009.

Carriers (Study 3003): ClinicalTrials.gov NCT00998764. Registered 16 October 2009.

**Electronic supplementary material:**

The online version of this article (doi:10.1186/s13195-016-0193-y) contains supplementary material, which is available to authorized users.

## Background

Alzheimer’s disease (AD) is characterized by a progressive decline in cognitive function and an increase in functional impairment [1–3]. Although the exact etiology of AD remains unknown, overproduction or inadequate clearance of accumulated amyloid-beta (Aβ) peptide is thought to be an essential component in its pathophysiology [1, 2, 4, 5]. One of the therapeutic approaches to halting disease progression in AD is passive immunotherapy with monoclonal antibodies (mAbs) directed against Aβ, which are thought to reduce Aβ accumulation and promote its clearance from the brain [4, 6]. This approach was shown to reduce cerebral Aβ deposits, improve behavioral measures, and reverse memory loss in animal models (reviewed in [2]), suggesting that removal of Aβ from the brain using mAbs may provide similar benefits in humans with AD. Bapineuzumab, a humanized mAb specific to the Aβ_1–42_ protein, has been evaluated in a total of four similarly designed phase 3 clinical trials for the treatment of patients with AD [6, 7]. As a result of phase 2 findings of amyloid-related imaging abnormalities with edema or effusions (ARIA-E), the bapineuzumab phase 3 development program stratified patients according to apolipoprotein E (ApoE) ε4 allele status (carriers or noncarriers) [8]. ARIA-E are detected as an increased signal intensity on FLAIR or other T2-weighted magnetic resonance imaging (MRI) sequences, and are thought to arise from an increase in permeability of brain capillaries to serum proteins, leading to extravasation of fluid into the extracellular space [9]. Incidence of ARIA-E was dose dependent, and 10 of the 12 cases were observed in ApoE ε4 carriers in the phase 2 study [8], which guided dose selection for the phase 3 trials in carriers and noncarriers. Studies 301 (noncarriers) and 302 (carriers) were conducted primarily in the United States, and Studies 3000 (noncarriers) and 3001 (carriers) were conducted at sites in more than 26 countries to evaluate the efficacy of intravenous (i.v.) bapineuzumab or placebo in patients with mild to moderate AD [7]. In the first two of these studies to be reported (Studies 301 and 302), no significant difference was found between the bapineuzumab and placebo groups in the primary clinical efficacy endpoints (Alzheimer’s Disease Assessment Scale—Cognitive Subscale (ADAS-Cog/11) and Disability Assessment Scale for Dementia (DAD)) [7]. ARIA-E were a primary safety finding in these studies in patients treated with bapineuzumab [7]. Based on the lack of clinical efficacy observed in the 301 and 302 studies, all ongoing bapineuzumab trials were terminated early by the sponsors in August 2012 [7]. Results from the terminated phase 3 parent studies (3000 and 3001) were presented in 2013 at the Clinical Trials on Alzheimer’s Disease symposium; consistent with the results of Studies 301 and 302, neither showed a clinical benefit of bapineuzumab therapy.

In this report, we detail findings from the 3002 and 3003 phase 3 extension studies of bapineuzumab in patients with AD who participated in the respective parent studies 3000 and 3001. The primary objective of the extension studies was to evaluate the long-term safety and tolerability of i.v.-administered bapineuzumab in subjects with AD, as it is not known how the rate of adverse events (AEs) and particularly ARIA-E evolves with the duration of therapy beyond the term of the phase 3 studies. The 3002 study evaluated ApoE ε4 allele noncarriers and the 3003 study evaluated ApoE ε4 allele carriers. Additional exploratory evaluations of cognitive, behavioral, and functional efficacy were conducted using validated assessment scales.

## Methods

### Study design

**Fig. 1:**
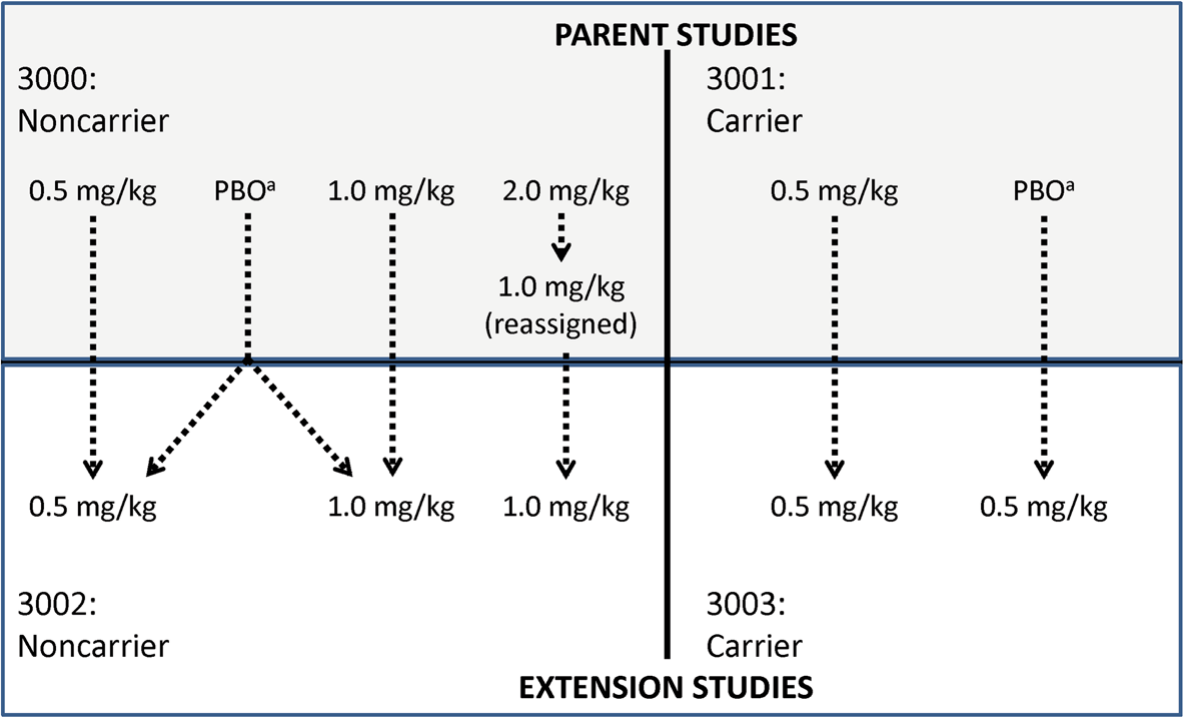
Schematic of treatment group assignments between parent and extension studies. ^a^Delayed-start treatment groups. *PBO* placebo

Both studies were phase 3, multicenter, long-term safety and tolerability extension trials of i.v. bapineuzumab once every 13 weeks in patients with AD. Prior to enrollment in either study, the protocols were reviewed and approved by the appropriate institutional review board or ethics committee, and all patients (or their legally acceptable representative) and patient caregivers provided informed consent. A complete list of all ethical bodies that approved the ApoE ε4 carrier and ApoE ε4 noncarrier studies are listed in Additional files 1 and 2, respectively. Both studies were conducted in accordance with principles set forth in the Declaration of Helsinki and according to good clinical practices established by the International Conference on Harmonisation. The 3002 study enrolled ApoE ε4 noncarriers who had completed Study 3000. If they received bapineuzumab in the main study, patients continued on bapineuzumab at the same dose (either 0.5 or 1.0 mg/kg); if they received placebo, patients were randomized to receive either bapineuzumab 0.5 mg/kg or 1.0 mg/kg. Patients who had originally received the discontinued bapineuzumab 2.0 mg/kg dose in the parent study were reassigned to 1.0 mg/kg during that study and continued to receive the 1.0 mg/kg dose in the extension study. All participants were aware that all patients were receiving bapineuzumab in the 3002 study, while treatment assignment from the 3000 study remained blinded to patients and to site staff, including the dose. The 3003 study enrolled ApoE ε4 carriers who had completed Study 3001. All patients were assigned to bapineuzumab 0.5 mg/kg whether they received bapineuzumab or placebo in the parent study. All participants were aware that all patients were receiving bapineuzumab 0.5 mg/kg, but treatment assignment from the 3001 study remained blinded to patients and to site staff. The transition of patients who received placebo in the parent studies to bapineuzumab treatment in the extension studies resulted in the formation of two subgroups: a bapineuzumab-to-bapineuzumab (bapineuzumab + bapineuzumab) “early start” group; and a placebo-to-bapineuzumab (placebo + bapineuzumab) “delayed start” group (Fig. 1). The initial planned duration of patient participation in the extension study was 2 years, which was extended to 4 years (208 weeks) per a protocol amendment dated July 2011. The first patient enrolled in the ApoE ε4 noncarrier (3002) study in February 2010 and in the ApoE ε4 carrier (3003) study in December 2009. Both studies were terminated in August 2012.

### Inclusion criteria

Patients had to have been enrolled in the parent studies (either 3000 or 3001) and completed all six infusions specified in the parent protocol; if the study drug had been suspended temporarily, the patients had to have completed all visits through week 78 and be eligible to resume investigational treatment. Patients met the inclusion criteria for AD in the parent studies (3000 and 3001) if they had: a diagnosis of probable AD according to the National Institute of Neurological and Communicative Disorders and Stroke–Alzheimer’s Disease and Related Disorders Association criteria; Rosen Modified Hachinski Ischemic Score ≤ 4; Mini-Mental State Examination (MMSE) score of 16–26, inclusive; and a screening brain MRI scan consistent with the diagnosis of AD. A brain MRI scan administered at week 71 of the parent study had to be available for local and central radiologic evaluation and remain consistent with a diagnosis of AD. Patients also had to have had a MMSE score ≥10 at screening (i.e., week 78 of the parent study). At each study center, the protocol and informed consent form and any amendments to these studies were reviewed and approved by a duly constituted institutional review board or independent ethics committee, and informed consent was obtained before any screening procedures specific to the extension studies were performed.

### Main exclusion criteria

Patients were excluded if they had any medical or psychiatric contraindication that, in the investigator’s opinion, could increase the risks associated with the patient’s continuation in or completion of the extension studies or might preclude an evaluation of response. Patients were also excluded if the brain MRI scan administered at week 71 in the parent study (3000 or 3001) was indicative of any significant abnormality, which for the extension studies included the following: four or more microhemorrhages (<10 mm), history or evidence of a single prior hemorrhage >1 cm^3^, two or more lacunar infarcts, evidence of a single prior infarct >1 cm^3^, and any abnormality detected in the MRI scan that was consistent with exclusion criteria in the parent studies.

### Concomitant AD medications

Patients who were receiving cholinesterase inhibitors or memantine for AD in the parent study (3000 or 3001) were allowed to continue at the same stable doses during the extension studies (3002 or 3003). Experimental medications for AD, all other experimental medications, and the use of herbal preparations containing ginkgo biloba were prohibited. Initiation of treatment with, or change in stable doses of, drugs with the potential to affect cognition, including cholinesterase inhibitors, memantine, over-the-counter drugs, and nutritional supplements, was prohibited unless medically indicated (e.g., side effects due to such drugs required dose reduction, or use of such drugs was temporarily stopped and restarted for medical reasons).

### Safety assessments

Both studies were continuously monitored by the same independent safety monitoring committee that functioned in the parent studies. The safety variables assessed comprised the incidence and severity of treatment-emergent AEs (TEAEs) throughout the study; safety laboratory variables, including clinical chemistry, hematology, and urinalysis (all analyzed centrally), and serum anti-bapineuzumab antibody; clinically important changes in vital signs, weight, and electrocardiograms (ECGs; with readings/interpretations performed centrally); and physical and neurological examinations. Brain MRI scans were radiologically assessed both centrally and locally. Both radiology reports were reviewed by the investigator prior to subsequent investigational product infusion. After the first year, MRI scan frequency was decreased from 13-week to 26-week intervals. Other safety variables included suicidality assessment and local reactions at the infusion site. ARIA-E, intraparenchymal brain hemorrhage, seizure, and deep vein thrombosis and/or pulmonary embolism (DVT/PE) were considered AEs of special circumstance, to be reported within 24 hours whether or not they were considered serious.

### Exploratory efficacy and biomarker assessments

The following clinical efficacy variables were assessed in both studies: ADAS-Cog/11, DAD, MMSE, and Neuropsychiatric Inventory (NPI). Three health outcomes measures were also explored: Dependence Scale, Resource Utilization in Dementia-Lite version, and Health Utilities Index. Biomarkers were assessed in a subset of patients in both studies, including change from baseline in cerebrospinal fluid (CSF), phosphorylated tau (p-tau), whole brain volume on volumetric MRI (vMRI), and [^11^C]-Pittsburgh Compound B positron emission tomography (PiB-PET), as in the parent studies.

## Results

### Patient disposition and exposure

Patient disposition and exposure in the 3002 and 3003 studies are presented in Table 1. In the ApoE ε4 carrier study, 506 patients were screened and 492 patients were enrolled; 216 patients had been randomized to placebo in the parent study (3001) and thus were identified as the placebo + bapineuzumab 0.5 mg/kg group (placebo + bapineuzumab 0.5), and 276 patients had originally received bapineuzumab and thus were identified as the bapineuzumab 0.5 mg/kg + bapineuzumab 0.5 mg/kg group (bapineuzumab 0.5 + bapineuzumab 0.5). The safety population was comprised of the 215 and 275 patients who were treated in the placebo + bapineuzumab 0.5 and bapineuzumab 0.5 + bapineuzumab 0.5 groups, respectively. In each treatment group, one patient was randomized but did not actually receive a dose of study medication, which accounts for the lower numbers in the safety population. The principle reason for withdrawal from treatment was sponsor decision; 76 % in the placebo + bapineuzumab 0.5 group and 79 % in the bapineuzumab 0.5 + bapineuzumab 0.5 group were withdrawn from treatment because the sponsors discontinued the study. Another 7 % and 4 % of patients, respectively, withdrew because of AEs. The mean number of person-years of exposure during this ApoE ε4 carrier extension study was 0.9 and 1.0, respectively.

**Table 1:** Patient disposition and exposure

	ApoE ε4 carriers		ApoE ε4 noncarriers			
	PBO + BAP 0.5	BAP 0.5 + BAP 0.5	PBO + BAP 0.5	BAP 0.5 + BAP 0.5	PBO + BAP 1.0	BAP 1.0 + BAP 1.0
Patients, *n* (%)						
Randomized	216 (100)	276 (100)	39 (100)	66 (100)	37 (100)	56 (100)
Treated	215 (99.5)	275 (99.6)	39 (100)	66 (100)	37 (100)	56 (100)
Completed^a^	1 (0.5)	2 (0.7)	0	0	0	0
Withdrawn from treatment and/or study^a^	214 (99.5)	273 (99.3)	39 (100)	66 (100)	37 (100)	56 (100)
Primary reason for withdrawal from treatment (safety analysis population), *n* (%)						
Unsatisfactory response-efficacy	3 (1.4)	6 (2.2)	1 (2.6)	1 (1.5)	2 (5.4)	2 (3.6)
Adverse event	16 (7.4)	11 (4.0)	2 (5.1)	5 (7.6)	2 (5.4)	6 (10.7)
Study termination	163 (75.8)	217 (78.9)	31 (79.5)	54 (81.8)	30 (81.1)	45 (80.4)
Subject request	19 (8.8)	25 (9.1)	1 (2.6)	4 (6.1)	3 (8.1)	3 (5.4)
Death	3 (1.4)	1 (0.4)	0	0	0	0
Recurrent episode of ARIA-E	1 (0.5)	0	0	0	0	0
All other reasons^b^	9 (4.2)	13 (4.7)	4 (10.2)	2 (3.0)	0	0
Person-years of study drug exposure^c^						
*N*	215	275	39	66	37	56
Mean (SD)	0.9 (0.58)	1.0 (0.58)	1.0 (0.53)	1.0 (0.61)	1.0 (5.4)	1.0 (5.4)
Median (range)	1 (0–3)	1 (0–3)	1 (0–2)	1 (0–3)	1 (0–2)	1 (0–2)

*ApoE* apolipoprotein E, *ARIA-E* amyloid-related imaging abnormalities with edema or effusions, *BAP* bapineuzumab, *PBO* placebo

^a^Percentage of treated patients

^b^Includes investigator request, protocol violation, failed to return, lost to follow-up, loss of caregiver, other

^c^Calculated as the number of days for each individual patient from the day of the first infusion of the extension study through either the day of the last infusion plus 137 days or the day of last study visit plus 1 day, whichever is shorter, divided by 365.25

In the ApoE ε4 noncarrier study, 209 patients were screened and 202 patients were enrolled. All patients withdrew from treatment, and none completed the study. Approximately 80 % of patients across treatment groups were withdrawn from treatment because the sponsor discontinued the study; across the groups, a total of 15 patients (7.4 %) withdrew because of AEs. The mean number of person-years of exposure during this extension study was 1.0 year in all groups. The number enrolled in the noncarrier study is notably lower than in the carrier study because enrollment in the parent study was much slower than in the parent carrier study. In addition, only 40 % of patients in the parent noncarrier study had completed the study at the time of early termination compared with over 60 % of patients in the parent carrier study. All enrolled patients were treated and included in the safety analysis population (*n* = 39 and *n* = 37 for the original placebo patients who were randomized to the placebo + bapineuzumab 0.5 and placebo + bapineuzumab 1.0 groups, respectively; *n* = 66 and *n* = 56 for the bapineuzumab 0.5 + bapineuzumab 0.5 and bapineuzumab 1.0 + bapineuzumab 1.0 groups, respectively; and an additional four patients who were transitioned from 2.0 mg/kg to 1.0 mg/kg in the parent noncarrier study who were included in the safety population but not in the other analyses).

### Demographics and baseline characteristics

Demographics and baseline characteristics for the safety population in both studies are presented in Tables 2 and 3. At the beginning of the extension study most patients were using cholinesterase inhibitors and/or memantine. Among ApoE ε4 carriers, 79 % had a single ApoE ε4 allele. The demographics at the beginning of the extension study were close to those observed at the baseline of the parent study across treatment groups. As expected, patients entering the extension study had a longer duration of AD than those beginning the parent study, approximately 4.4 years vs 2.9 years; mean MMSE scores were lower at the beginning of the extension study compared with baseline MMSE in the parent study.

**Table 2:** Patient demographics and baseline characteristics at study entry (parent study safety population)

	3001 ApoE ε4 carrier study		3000 ApoE ε4 noncarrier study		
	PBO (*n* = 215)	BAP 0.5 (*n* = 275)	PBO (*n* = 76)	BAP 0.5 (*n* = 66)	BAP 1.0 (*n* = 56)
Mean age (years)	69.8	70.6	67.3	69.8	68.9
Female^a^ (%)	62.3	67.6	61.8	53.0	62.5
White^a^ (%)	80.9	75.3	78.9	74.2	66.1
Asian (%)	18.1	22.9	21.1	25.8	32.1
Mean duration of AD (years)	2.89	2.98	2.73	2.85	2.87
Mean baseline MMSE	21.3	21.4	20.2	20.8	20.6
Current AChEI and/or memantine use, *n* (%)					
Yes	199 (92.6)	255 (92.7)	70 (92.1)	56 (84.8)	54 (96.4)
No	16 (7.4)	20 (7.3)	6 (7.9)	10 (15.2)	2 (3.6)
ApoE ε4 allele count, *n* (%)					
1	171 (79.5)	217 (78.9)	NA	NA	NA
2	44 (20.5)	58 (21.1)	NA	NA	NA

*AChEI* acetylcholinesterase inhibitor, *AD* Alzheimer’s disease, *ApoE* apolipoprotein E, *BAP* bapineuzumab, *MMSE* Mini-Mental State Examination, *NA* not applicable, *PBO* placebo

^a^A few patients may have been misclassified at baseline, causing discrepancy with Table 3: one patient was classified as male at parent baseline and female at extension baseline; three patients were classified as white at parent baseline and Asian or “other” at extension baseline

**Table 3:** Patient demographics and baseline characteristics from extension study baseline

	3003 ApoE ε4 carrier study		3002 ApoE ε4 noncarrier study			
	PBO + BAP 0.5 (*n* = 215)	BAP 0.5 + BAP 0.5 (*n* = 275)	PBO + BAP 0.5 (*n* = 39)	PBO + BAP 1.0 (*n* = 37)	BAP 0.5 + BAP 0.5 (*n* = 66)	BAP 1.0 + BAP 1.0 (*n* = 56)
Mean age (years)	71.4	72.1	68.7	68.9	71.4	70.6
Female^a^ (%)	62.8	67.6	64.1	59.5	53.0	62.5
White^a^ (%)	79.5	75.6	76.9	81.1	74.2	66.1
Asian (%)	18.1	22.9	23.1	18.9	25.8	32.1
Mean duration of AD (years)	4.49	4.58	4.16	4.50	4.44	4.46
Mean baseline MMSE	19.0^b^	19.2	18.6^c^	17.1	19.1	18.4
Current AChEI and/or memantine use, *n* (%)						
Yes	193 (89.8)	242 (88.0)	34 (87.2)	31 (83.8)	51 (77.3)	53 (94.6)
No	22 (10.2)	33 (12.0)	5 (12.8)	6 (16.2)	15 (22.7)	3 (5.4)

*AChEI* acetylcholinesterase inhibitor, *AD* Alzheimer’s disease, *ApoE* apolipoprotein E, *BAP* bapineuzumab, *MMSE* Mini-Mental State Examination, *PBO* placebo

^a^A few patients may have been misclassified at baseline, causing discrepancy with Table 2: one patient was classified as male at parent baseline and female at extension baseline; three patients were classified as white at parent baseline and Asian or “other” at extension baseline

^b^ *n* = 212

^c^ *n* = 38

In ApoE ε4 carriers (Study 3003), the mean age at the beginning of the extension study (Table 3) was similar between groups. Most patients were white; 20.8 % were Asian. More than half were female and the mean duration of AD was 4.49 and 4.58 years in the placebo + bapineuzumab and bapineuzumab + bapineuzumab groups, respectively. Baseline MMSE scores were 19.0 and 19.2, respectively, at the beginning of the extension study.

In ApoE ε4 noncarriers (Study 3002), the mean age at the beginning of the extension study was also similar between groups (Table 3). Most patients were white, 25.2 % were Asian, and more than half of the patients were female. Across treatment groups, the mean duration of AD was 4.38 years at the beginning of the extension study, and the mean MMSE score was 18.4.

### Safety

Overall, the percentages of TEAEs, serious AEs (SAEs), TEAEs leading to early termination from the study, TEAEs leading to early termination from treatment, and TEAEs leading to dose reduction or temporary discontinuation were similar for patients in the carrier (3003) and noncarrier (3002) studies (Table 4). In the bapineuzumab 2.0 + bapineuzumab 1.0 group (not shown in the tables), no SAEs were reported and eight TEAEs were reported in three patients (75 %), all of which were of mild intensity and none of these events were considered by the investigator to be related to study treatment.

**Table 4:** Overview of treatment-emergent adverse events in the safety population

	3003 ApoE ε4 carrier study		3002 ApoE ε4 noncarrier study			
	PBO + BAP 0.5 (*n* = 215)	BAP 0.5 + BAP 0.5 (*n* = 275)	PBO + BAP 0.5 (*n* = 39)	PBO + BAP 1.0 (*n* = 37)	BAP 0.5 + BAP 0.5 (*n* = 66)	BAP 1.0 + BAP 1.0 (*n* = 56)
Any TEAE	152 (70.7)	184 (66.9)	32 (82.1)	25 (67.6)	48 (72.7)	36 (64.3)
Any SAE	35 (16.3)	33 (12.0)	6 (15.4)	1 (2.7)	10 (15.2)	11 (19.6)
TEAE leading to treatment discontinuation	18 (8.4)	10 (3.6)	2 (5.1)	2 (5.4)	5 (7.6)	6 (10.7)
TEAE leading to study discontinuation	14 (6.5)	10 (3.6)	2 (5.1)	1 (2.7)	1 (1.5)	1 (1.8)
TEAE leading to dose reduction or temporary discontinuation	18 (8.4)	10 (3.6)	2 (5.1)	3 (8.1)	1 (1.5)	4 (7.1)

Data presented as *n* (%)

*ApoE* apolipoprotein E, *BAP* bapineuzumab, *PBO* placebo, *SAE* serious adverse event, *TEAE* treatment-emergent adverse event

#### TEAEs

TEAEs for the carrier and noncarrier studies are shown in Tables 5 and 6, respectively. In ApoE ε4 carriers (Study 3003), TEAEs were reported in 152 patients (70.7 %) in the placebo + bapineuzumab 0.5 group and 184 patients (66.9 %) in the bapineuzumab 0.5 + bapineuzumab 0.5 group. The most common TEAEs occurring in ≥5 % regardless of causality were ARIA-E, cerebral microhemorrhage, headache, diarrhea, urinary tract infection, and anxiety. ARIA-E and cerebral microhemorrhage had different reporting requirements, so patients may have had either one or both.

**Table 5:** Treatment-emergent adverse events (≥5 % in either group), safety population: carrier study

Event	PBO + BAP 0.5 (*n* = 215)	BAP 0.5 + BAP 0.5 (*n* = 275)
ARIA-E (vasogenic cerebral edema)	23 (10.7)	10 (3.6)
Cerebral microhemorrhage	20 (9.3)	15 (5.5)
Headache	16 (7.4)	8 (2.9)
Diarrhea	12 (5.6)	10 (3.6)
Urinary tract infection	12 (5.6)	9 (3.3)
Anxiety	11 (5.1)	7 (2.5)

Data presented as *n* (%)

*ARIA-E* amyloid-related imaging abnormalities with edema or effusions, *BAP* bapineuzumab, *PBO* placebo

**Table 6:** Treatment-emergent adverse events (≥5 % in any group), safety population: noncarrier study

Event	PBO + BAP 0.5 (*n* = 39)	BAP 0.5 + BAP 0.5 (*n* = 66)	PBO + BAP 1.0 (*n* = 37)	BAP 1.0 + BAP 1.0 (*n* = 56)
ARIA-E (vasogenic cerebral edema)	3 (7.7)	2 (3.0)	6 (16.2)	3 (5.4)
Urinary tract infection	2 (5.1)	7 (10.6)	1 (2.7)	0
Headache	1 (2.6)	1 (1.5)	3 (8.1)	2 (3.6)
Gastroenteritis	0	0	3 (8.1)	0
Nasopharyngitis	3 (7.7)	1 (1.5)	1 (2.7)	2 (3.6)
Delusion	3 (7.7)	3 (4.5)	0	0
Gait disturbance	3 (7.7)	0	1 (2.7)	0
Fall	0	3 (4.5)	2 (5.4)	4 (7.1)
Dizziness	1 (2.6)	4 (6.1)	0	0
Cerebral microhemorrhage	0	1 (1.5)	0	3 (5.4)
Cognitive disorder	1 (2.6)	2 (3.0)	2 (5.4)	3 (5.4)
Depression	0	0	2 (5.4)	0
Subdural hematoma	0	0	0	3 (5.4)
Aggression	2 (5.1)	0	0	0
Anemia	2 (5.1)	1 (1.5)	0	0
Cough	2 (5.1)	0	0	2 (3.6)
Nausea	2 (5.1)	0	0	0

Data presented as *n* (%)

*ARIA-E* amyloid-related imaging abnormalities with edema or effusions, *BAP* bapineuzumab, *PBO* placebo

In ApoE ε4 noncarriers (Study 3002), TEAEs were reported in 32 (82.1 %) of those in the placebo + bapineuzumab 0.5 group, 25 of those (67.6 %) in the placebo + bapineuzumab 1.0 group, 48 of those (72.7 %) in the bapineuzumab 0.5 + bapineuzumab 0.5 group, and 36 of those (64.3 %) in the bapineuzumab 1.0 + bapineuzumab 1.0 group. The most common TEAEs occurring in ≥5 % regardless of causality were ARIA-E, urinary tract infection, headache, gastroenteritis, nasopharyngitis, delusion, gait disturbance, and fall (Table 6).

#### TEAEs leading to treatment discontinuation or study discontinuation

Patients who had to discontinue use of study medication were encouraged to continue to attend scheduled study visits and undergo applicable procedures if feasible. In ApoE ε4 carriers, TEAEs led to treatment discontinuation in <10 % of patients and study discontinuation in <7 % of patients in either group; these numbers were higher in the placebo + bapineuzumab 0.5 group than in the bapineuzumab 0.5 + bapineuzumab 0.5 group (Table 4).

In ApoE ε4 noncarriers, TEAEs led to treatment discontinuation in <11 % of patients in any treatment group, and the numbers were lowest in the placebo + bapineuzumab groups and highest in the bapineuzumab 1.0 + bapineuzumab 1.0 group. TEAEs leading to study discontinuation occurred in <6 % of patients in any treatment group, and the numbers were higher in the placebo + bapineuzumab groups (Table 4).

#### SAEs and deaths

In ApoE ε4 carriers, there were five deaths (2.3 %) in the placebo + bapineuzumab 0.5 group (cardiac failure, metastases to lymph nodes, ovarian cancer, pancreatic carcinoma, subarachnoid hemorrhage) and two deaths (0.7 %) in the bapineuzumab 0.5 + bapineuzumab 0.5 group (cardiac failure congestive and cardiomyopathy). Of these, one death was due to a TEAE assessed as related to bapineuzumab treatment by the study investigator (subarachnoid hemorrhage in the placebo + bapineuzumab 0.5 group). The corresponding rates of treatment-emergent SAEs in these treatment groups were 16.3 % and 12.0 %, respectively.

In ApoE ε4 noncarriers, there were no deaths. Treatment-emergent SAEs occurred in six patients (15.4 %) in the placebo + bapineuzumab 0.5 group, 10 patients (15.2 %) in the bapineuzumab 0.5 + bapineuzumab 0.5 group, one patient (2.7 %) in the placebo + bapineuzumab 1.0 group, and 11 patients (19.6 %) in the bapineuzumab 1.0 + bapineuzumab 1.0 group.

#### AEs of special circumstance

In ApoE ε4 carriers (Study 3003), ARIA-E was reported as a TEAE in 23 patients (10.7 %) in the placebo + bapineuzumab 0.5 group and 10 patients (3.6 %) in the bapineuzumab 0.5 + bapineuzumab 0.5 group. ARIA-E was asymptomatic in 16 (7.4 %) and nine (3.3 %) patients in the two treatment groups, respectively. ARIA-E was reported as a treatment-emergent SAE in six patients (2.8 %) in the placebo + bapineuzumab 0.5 group and two patients (0.7 %) in the bapineuzumab 0.5 + bapineuzumab 0.5 group. In the ApoE ε4 noncarriers (Study 3002), ARIA-E was reported as a TEAE in three patients (7.7 %) in the placebo + bapineuzumab 0.5 group, two patients (3.0 %) in the bapineuzumab 0.5 + bapineuzumab 0.5 group, six patients (16.2 %) in the placebo + bapineuzumab 1.0 group, and three patients (5.4 %) in the bapineuzumab 1.0 + bapineuzumab 1.0 group; overall, the percentage was greater in the placebo + bapineuzumab groups (11.8 %) than in the bapineuzumab + bapineuzumab groups (4.1 %). All were asymptomatic except for one patient in the placebo + bapineuzumab 0.5 group. ARIA-E was reported as a treatment-emergent SAE in two patients, one patient in each of the placebo + bapineuzumab groups, and no patients in either of the bapineuzumab + bapineuzumab groups.

In the carrier study, intracranial hemorrhage (including intraparenchymal brain hemorrhage, subdural, intraventricular, and subarachnoid bleeding, but excluding microhemorrhages or larger hemosiderin deposits) was reported as a TEAE in six patients (2.8 %) in the placebo + bapineuzumab 0.5 group and none in the bapineuzumab 0.5 + bapineuzumab 0.5 group. One cerebral hemorrhage and two subarachnoid hemorrhages were reported as treatment-emergent SAEs; one subarachnoid hemorrhage was fatal. In the noncarrier study, intracranial hemorrhage was reported as a TEAE in one patient in the bapineuzumab 0.5 + bapineuzumab 0.5 group, three patients in the bapineuzumab 1.0 + bapineuzumab 1.0 group, and no patients in either of the placebo + bapineuzumab groups. One subarachnoid hemorrhage in the bapineuzumab 0.5 + bapineuzumab 0.5 group was reported as a treatment-emergent SAE.

In the carrier study, seizure/convulsion was reported in four patients (1.9 %) in the placebo + bapineuzumab 0.5 group and three patients (1.1 %) in the bapineuzumab 0.5 + bapineuzumab 0.5 group. In the noncarrier study, seizure/convulsion was reported in one patient (2.6 %) in the placebo + bapineuzumab 0.5 group, one patient (1.5 %) in the bapineuzumab 0.5 + bapineuzumab 0.5 group, one patient (2.7 %) in the placebo + bapineuzumab 1.0 group, and no patients in the bapineuzumab 1.0 + bapineuzumab 1.0 group.

DVT/PE was reported in one patient in the placebo + bapineuzumab 0.5 group in the carrier study and two patients in the bapineuzumab 0.5 + bapineuzumab 0.5 group in the noncarrier study.

### Exploratory assessments

Overall, the changes in scores were similar between the dose groups across cognitive, functional, and behavioral measures in both studies. None of the health outcomes measures showed any significant differences in either study. The least-squares (LS) mean change in ADAS-Cog/11 and DAD scores from parent study baseline to extension study week 52 were not significantly different between treatment groups in either the ApoE ε4 carriers (Study 3003) or ApoE ε4 noncarriers (Study 3002) (Tables 7 and 8). In the carrier study, the between-group difference in ADAS-Cog/11 score change from baseline was 0.0 (*P* = 0.996) and the between-group difference in DAD score change from baseline was –1.75 (*P* = 0.412). NPI and MMSE scores were not significantly different in changes from baseline (Table 7). Similarly, in the noncarrier study the between-group difference in LS mean change in ADAS-Cog/11 score between the placebo + bapineuzumab 0.5 and bapineuzumab 0.5 + bapineuzumab 0.5 groups was –2.05 (*P* = 0.345); and between the placebo + bapineuzumab 1.0 and bapineuzumab 1.0 + bapineuzumab 1.0 groups, the difference was –0.22 (*P* = 0.922). The between-group difference in LS mean change in DAD scores between the placebo + bapineuzumab 0.5 and bapineuzumab 0.5 + bapineuzumab 0.5 groups was 1.53 (*P* = 0.733); and between the placebo + bapineuzumab 1.0 and bapineuzumab 1.0 + bapineuzumab 1.0 groups, the difference was –2.81 (*P* = 0.550). NPI and MMSE scores were also not significantly different in changes from baseline (Table 8).

**Table 7:** Carrier study exploratory clinical efficacy assessments: change from parent study baseline to extension week 52

	3003 ApoE ε4 carrier study	
	PBO + BAP 0.5 (*n* = 199)	BAP 0.5 + BAP 0.5 (*n* = 256)
ADAS-Cog, LS mean (SE)	10.12 (0.75)	10.11 (0.63)
LS mean difference	0.00; *P* = 0.996	
DAD, LS mean (SE)	–20.96 (1.65)	–22.72 (1.36)
LS mean difference	–1.75; *P* = 0.412	
NPI, LS mean (SE)	4.32 (0.96)	3.52 (0.80)
LS mean difference	–0.80; *P* = 0.524	
MMSE^a^, LS mean (SE)	–4.44 (0.26)	–4.26 (0.21)
LS mean difference	0.18; *P* = 0.598	

*ADAS-Cog* Alzheimer’s Disease Assessment Scale—Cognitive Subscale, *ApoE* apolipoprotein E, *BAP* bapineuzumab, *DAD* Disability Assessment Scale for Dementia, *LS* least squares, *MMSE* Mini-Mental State Examination, *NPI* Neuropsychiatric Inventory, *PBO* placebo, *SE* standard error of the mean

^a^Difference in MMSE score was between parent study baseline and extension study week 45

**Table 8:** Noncarrier study exploratory clinical efficacy assessments: change from parent study baseline to extension week 52

	3002 ApoE ε4 noncarrier study			
	PBO + BAP 0.5 (*n* = 38)	BAP 0.5 + BAP 0.5 (*n* = 62)	PBO + BAP 1.0 (*n* = 33)	BAP 1.0 + BAP 1.0 (*n* = 53)
ADAS-Cog, LS mean (SE)	12.44 (1.70)	10.38 (1.35)	10.54 (1.82)	10.31 (1.36)
LS mean difference	–2.05; *P* = 0.345		–0.22; *P* = 0.922	
DAD, LS mean (SE)	–29.52 (3.48)	–28.00 (2.77)	–20.41 (3.74)	–23.21 (2.80)
LS mean difference	1.53; *P* = 0.733		–2.81; *P* = 0.550	
NPI, LS mean (SE)	4.99 (2.60)	–0.01 (2.05)	4.23 (2.80)	6.57 (2.08)
LS mean difference	–4.99; *P* = 0.133		2.34; *P* = 0.505	
MMSE^a^, LS mean (SE)	–4.86 (0.56)	–4.13 (0.44)	–4.63 (0.58)	–4.51 (0.46)
LS mean difference	0.73; *P* = 0.309		0.12; *P* = 0.872	

*ADAS-Cog* Alzheimer’s Disease Assessment Scale—Cognitive Subscale, *ApoE* apolipoprotein E, *BAP* bapineuzumab, *DAD* Disability Assessment Scale for Dementia, *LS* least squares, *MMSE* Mini-Mental State Examination, *NPI* Neuropsychiatric Inventory, *PBO* placebo, *SE* standard error of the mean

^a^Difference in MMSE score was between parent study baseline and extension study week 45

In both the ApoE ε4 carrier and noncarrier studies, only a very limited amount of data was obtained for the biomarker studies because of the premature discontinuation of the study by the sponsor. The PiB-PET analysis population patients (who had standardized uptake value ratio ≥ 1.35 at baseline in the parent study and a postbaseline assessment in the extension study) comprised one patient in the carrier study and two patients in the noncarrier study. For the CSF biomarker substudy, 15 patients were enrolled in the carrier study and 14 patients in the noncarrier study; no significant differences in CSF p-tau were observed in either study. In the vMRI substudy, 26 patients in the combined placebo + bapineuzumab group and 35 patients in the combined bapineuzumab + bapineuzumab group in the noncarrier study had assessments of whole brain volume at extension study baseline and week 45. The LS mean change and standard error of the mean (SE) were –30.2 (8.1) in the placebo + bapineuzumab group and –12.6 (6.8) in the bapineuzumab + bapineuzumab group, for a difference of 17.5 (*P* = 0.111). Fifty-one patients in the placebo + bapineuzumab group and 78 patients in the bapineuzumab + bapineuzumab group in the carrier study had assessments of whole brain volume at extension study baseline and week 45. The LS mean change and SE were –60.28 (21.94) in the placebo + bapineuzumab group and –38.67 (17.88) in the bapineuzumab + bapineuzumab group, for a difference of 21.61 (*P* = 0.447).

## Discussion

The present studies examined the safety of long-term treatment with bapineuzumab in patients with AD who participated in the parent studies 3000 and 3001. As such, they are among the first studies to report the safety of regular dosing of a mAb against Aβ over a period of approximately 3.5 years.

### Safety

Infusion of bapineuzumab 0.5 or 1.0 mg/kg every 13 weeks for up to 3.5 years was generally well tolerated, with a safety and tolerability profile that was similar to that observed in previous studies. The long-term safety and tolerability profile was generally similar in ApoE ε4 noncarriers and carriers in the extension studies, except for a higher incidence proportion of treatment-emergent death in carriers. There was a higher incidence proportion of ARIA-E (vasogenic edema) observed with onset during the study in both noncarrier and carrier patients who first received bapineuzumab in the extension studies than in those who had received bapineuzumab from the beginning of the parent studies. This finding further supports the observations during the double-blind studies that ARIA-E tends to occur early in the course of bapineuzumab exposure (most often between infusions 1 and 3) [7], with a decline in the incidence of ARIA-E over time with continued bapineuzumab exposure during the double-blind studies. This finding is consistent with a reduced risk of ARIA-E after longer exposure. However, in patients who continued on bapineuzumab in both extension studies, ARIA-E was still seen after the sixth dose in 3.6 % of carriers and 4.1 % of noncarriers, and the latest ARIA-E occurred after the twelfth dose of bapineuzumab. There was also evidence of a dose effect for ARIA-E in the noncarrier study, consistent with previous studies, which demonstrated that the risk of ARIA-E increased with bapineuzumab dose [7].

### Efficacy

To date, a clinical benefit of amyloid-targeted immunotherapy with bapineuzumab or any other mAb has not been observed for patients with AD [7, 10]. Recently reported findings from pivotal phase 3 studies of bapineuzumab in patients with mild to moderate AD failed to show a significant difference in clinical endpoints of ADAS-Cog/11 and DAD scores or other clinical endpoints [7]. Among ApoE ε4 carriers (but not noncarriers), bapineuzumab was associated with reduced CSF p-tau concentrations, a marker of neurodegeneration. Carriers also showed a decrease in amyloid accumulation based on PiB-PET findings. Similarly, another mAb, solanezumab, which preferentially binds to soluble forms of Aβ, also failed to improve clinical parameters in patients with mild to moderate AD in the phase 3 trials [10]. Gantenerumab, a fully human mAb that binds to two regions of monomeric and fibrillar Aβ, reduced brain amyloid burden in a multiple ascending dose study, findings that were not correlated with cognitive measures [11]. In the long-term extension studies reported here, the biomarker subpopulations were too small to support substantial analyses. The results for ADAS-Cog/11 and DAD were similar for carriers and noncarriers, showing declining cognition (ADAS-Cog/11) and function (DAD) consistent with AD progression over time [12]. They were also similar between the two dose groups in the noncarrier study, suggesting no adverse effect of the higher dose on cognitive measures. Despite the lack of efficacy in patients with mild to moderate AD, it has been speculated that the earlier use of these therapies in mild AD, or in asymptomatic patients with Aβ accumulation, may be of benefit [7, 10, 13]. It is unknown whether the use of immunotherapy earlier in the disease process, perhaps before the onset of disability (early AD or pre-AD), will result in clinical benefit [7, 10, 13], and whether such a population can be identified. These studies provide some evidence to support the safety and tolerability of the long-term administration of anti-Aβ therapy, which would be useful when treating earlier in the disease process.

### Study limitations

The studies described herein have several limitations, including the lack of a placebo control in the extension groups and the fact that they were not fully randomized. Early discontinuation of the trials affected the number of patients available for the exploratory clinical efficacy and health outcomes analyses, as well as the biomarker analyses (CSF, PiB-PET, vMRI).

## Conclusions

The i.v. infusion of bapineuzumab 0.5 mg/kg to ApoE ε4 carriers or of bapineuzumab 0.5 mg/kg or 1.0 mg/kg to ApoE ε4 noncarriers every 13 weeks for up to 3.5 years was generally well tolerated. The safety and tolerability profile of bapineuzumab was similar to that observed in previous studies of bapineuzumab, and no new or unexpected safety concerns were identified.

## Abbreviations

AD, Alzheimer’s disease; ADAS-Cog/11, Alzheimer’s Disease Assessment Scale—Cognitive Subscale; AE, adverse event; ApoE, apolipoprotein E; ARIA-E, amyloid-related imaging abnormalities with edema or effusions; Aβ, amyloid beta; BAP, bapineuzumab; CSF, cerebrospinal fluid; DAD, Disability Assessment Scale for Dementia; DVT/PE, deep vein thrombosis and/or pulmonary embolism; i.v., intravenous; LS, least squares; mAb, monoclonal antibody; MMSE, Mini-Mental State Examination; MRI, magnetic resonance imaging; NPI, Neuropsychiatric Inventory; PBO, placebo; PiB-PET, Pittsburgh Compound B positron emission tomography; p-tau, phosphorylated tau; SAE, serious adverse event; SE, standard error of the mean; TEAE, treatment-emergent adverse event; vMRI, volumetric magnetic resonance imaging.

## Additional files

Additional file 1: is a list of independent ethics committees or institutional review boards that approved the ApoE ε4 carrier studies. (PDF 114 kb) Additional file 2: is a list of independent ethics committees or institutional review boards that approved the ApoE ε4 noncarrier studies. (PDF 124 kb)

## References

[1] Liu CC, Kanekiyo T, Xu H, Bu G (2013). Apolipoprotein E and Alzheimer disease: risk, mechanisms and therapy. Nat Rev Neurol.

[2] Madeo J, Frieri M (2013). Alzheimer’s disease and immunotherapy. Aging Dis..

[3] Singh S, Kushwah AS, Singh R, Farswan M, Kaur R (2012). Current therapeutic strategy in Alzheimer’s disease. Eur Rev Med Pharmacol Sci..

[4] Lemere CA (2013). Immunotherapy for Alzheimer’s disease: hoops and hurdles. Mol Neurodegener..

[5] Alzheimer’s Association. 2013 Alzheimer’s disease facts and figures. 2013. http://www.alz.org/downloads/facts_figures_2013.pdf. Accessed 8 Dec 2015.

[6] Tayeb HO, Murray ED, Price BH, Tarazi FI (2013). Bapineuzumab and solanezumab for Alzheimer’s disease: is the “amyloid cascade hypothesis” still alive?. Expert Opin Biol Ther..

[7] Salloway S, Sperling R, Fox NC, Blennow K, Klunk W, Raskind M (2014). Bapineuzumab 301 and 302 Clinical Trial Investigators. Two phase 3 trials of bapineuzumab in mild-to-moderate Alzheimer’s disease. N Engl J Med.

[8] Salloway S, Sperling R, Gilman S, Fox NC, Blennow K, Raskind M (2009). Bapineuzumab 201 Clinical Trial Investigators. A phase 2 multiple ascending dose trial of bapineuzumab in mild to moderate Alzheimer disease. Neurology.

[9] Sperling RA, Jack CR, Black SE, Frosch MP, Greenberg SM, Hyman BT (2011). Amyloid-related imaging abnormalities in amyloid-modifying therapeutic trials: recommendations from the Alzheimer's Association Research Roundtable Workgroup. Alzheimers Dement..

[10] Doody RS, Thomas RG, Farlow M, Iwatsubo T, Vellas B, Joffe S (2014). Alzheimer’s Disease Cooperative Study Steering Committee; Solanezumab Study Group: phase 3 trials of solanezumab for mild-to-moderate Alzheimer’s disease. N Engl J Med..

[11] Panza F, Solfrizzi V, Imbimbo BP, Giannini M, Santamato A, Seripa D (2014). Efficacy and safety studies of gantenerumab in patients with Alzheimer's disease. Expert Rev Neurother..

[12] Blennow K, de Leon MJ, Zetterberg H (2006). Alzheimer’s disease. Lancet..

[13] Blennow K, Hampel H, Zetterberg H (2014). Biomarkers in amyloid-β immunotherapy trials in Alzheimer’s disease. Neuropsychopharmacology..

