# Supplementary material for: Long-term safety and tolerability of bapineuzumab in patients with Alzheimer’s disease in two phase 3 extension studies
Source: Alzheimers Res Ther. 2016 Jun 23;8:24. doi: 10.1186/s13195-016-0193-y (PMC4918115; doi:10.1186/s13195-016-0193-y)
Supplement: Additional file 1: — is a list of independent ethics committees or institutional review boards that approved the ApoE ε4 carrier studies. (PDF 114 kb) [file 13195_2016_193_MOESM1_ESM.pdf]

16.1.3 List of Independent Ethics Committees or Institutional Review Boards

A list of the following is included:

- The List of Independent Ethics Committees, sorted by country and site, for sites that screened subjects.

Independent Ethics Committees

Site number	Name and Address of Committee
**Argentina**	
173	Comite de Etica Independiente en Investigacion Clinica Larrea 1381- 3°A Buenos Aires C1117ABK
**Australia**	
52<br>56	Northern Sydney / Central Coast Area Health Service HREC Human Research Ethics Committee, Royal North Shore Hospital, Level 2 Building 51, Pacific Hwy, St Leonards, NSW 2065
53	Austin Health Human Research Ethics Committee 145 Studley Road Heidelberg, Vic 3084
55	Hollywood Private Hospital Research Ethics Committee 101 Monash Avenue, Nedlands, WA 6009
57	Central Northern Adelaide Health Service Ethics of Human Research Committee (TQEH & LMH) The Queen Elizabeth Hospital, 28 Woodville Road, Woodville South, SA 5011
58	Royal Adelaide Hospital Research Ethics Committee Royal Adelaide Hospital, Level 3, Hanson Centre, North Terrace, Adelaide, SA 5000
**Belgium**	
46	Commissie Medische Ethiek Toetsingscommissie, Herestraat 49 3000 Leuven

Site number	Name and Address of Committee
**Belgium**	
47<br>48<br>265<br>272	Commissie Medische Ethiek van de Universitaire<br>Ziekenhuizen KU Campus Gasthuisberg E330<br>Herestraat 49,<br>Leuven 3000
**Chile**	
126	Comite Etico Cientifico, Servicio de Salud<br>Metropolitano Oriente Av. Salvador 364<br>Providencia,<br>Santiago, RM 7500922
**Finland**	
3<br>6	Pohjois-Savon sairaanhoitopiirin ky Tutkimuseettinen toimikunta<br>Rakennus 10 (4. krs)<br>PL 1777,<br>Kuopio 70211

Site number	Name and Address of Committee
**France**	
109<br>110<br>111<br>112<br>114<br>115<br>116<br>117<br>119<br>120<br>121<br>122<br>123<br>124<br>125<br>157<br>158<br>291	CPP Ile de France 3 Hôpital Tarnier Cochin<br>89, rue d'Assas,<br>Paris 75006
**Italy**	
29	Comitato Etico Indipendente dell'IRCCS Fondazione S. Lucia di Roma<br>Via Ardeatina, 306,<br>Roma 00179
33	Comitato Etico della Fondazione IRCCS Istituto Neurologico Carlo Besta di Milano<br>Via Celoria, 11,<br>Milano 20133
34	Comitato Etico Comitato Etico dell'Azienda Ospedaliero Universitaria<br>Ospedali Riuniti Umberto I - G.M. Lancisi - G. Salesi di ancona<br>Via Conca, 71<br>Ancona 60126

Site number	Name and Address of Committee
**Italy**	
41	Comitato Etico dell'Azienda Ospedaliero Universitaria Policlinico Vittorio Emanuele di Catania Via Santa Sofia, 78<br>Catania 95123
**Japan**	
177	National Hospital Organization Kokura Medical Center 10-1 Harugaoka<br>Kokuraminami-ku<br>Kitakyusyu-shi,<br>Fukuoka 802-8533
179	National Hospital Organization Minami-Okayama Medical Center IRB 4066<br>Hayashima, Hayashima-cho,<br>Tukubo-Gun, Okayama 701-0304
180<br>204	Juntendo University Hospital IRB 3-1-3<br>Hongo<br>Bunkyo-ku,<br>Tokyo 113-8431
181	National Hospital Organization Niigata National Hospital IRB 3-52 Akasaka-cho<br>kashiwazaki,<br>Niigata 945-8585
182	National Hospital Organization Chiba-East Hospital 673<br>Nitona-chou<br>Chuou-ku<br>Chiba,<br>Chiba 260-8712
183	Kagawa University Hospital IRB 1750-1<br>Ikenobe<br>Miki-cho<br>Kita-gun,<br>Kagawa 761-0793

Site number	Name and Address of Committee
**Japan**	
185	Osaka City University Hospital 1-5-7,<br>Asahi-machi,<br>Abeno-ku,<br>Osaka,<br>Osaka 545-8586
186	Juntendo Tokyo Koto Geriatric Medical Center IRB<br>3-3-20<br>Shinsuna<br>Koto-ku,<br>Tokyo 136-0075
187	Tokyo Medical University Hospital 6-7-1<br>Nishi-Shinjuku<br>Shinjuku-ku,<br>Tokyo 160-0023
188	Rakuwakai Otowa Hospital IRB 2 Chinji-cho,<br>Otowa,<br>Yamashina-ku<br>Kyoto-shi,<br>Kyoto 607-8062
189	Okayama University Hospital IRB 2-5-1 Shikata-cho,<br>Kita-ku<br>Okayama,<br>Okayama 700-8558
190	National Hospital Organization Minami-Kyoto<br>Hospital IRB 11 Ashihara, Naka,<br>Jouyou-shi,<br>Kyoto 610-0113
191	National Hospital Organization Hiroshima-nishi<br>Medical Center 4-1-1<br>Kuba<br>Otake city,<br>Hiroshima 739-0696

Site number	Name and Address of Committee
**Japan**	
192	National Hospital Organization Tokyo National Hospital IRB 3-1-1 Takeoka,<br>Kiyose-shi,<br>Tokyo 204-8585
193	National Hospital Organization Maizuru Medical Center IRB 2410 Aza Yukinaga,<br>Maizuru,<br>Kyoto 625-8502
194	Kansai Medical University Takii Hospital 10-15 Fujizono-cho<br>Moriguchi,<br>Osaka 570-8507
195	Gunma University Hospital 3-39-15<br>Showa-machi<br>Maebashi,<br>Gunma 371-8511
196	Kobe University Hospital IRB 7-5-2<br>kusunoki-cho, Chuo-ku<br>Kobe,<br>Hyogo 650-0017
197	Nagoya City University Hospital IRB Nagoya City University Hospital IRB<br>1<br>Kawasumi Mizuho-cho,<br>Mizuho-ku, Nagoya,<br>Aichi 467-8602
200	Tokyo Medical University Hachioji Medical Center 1163<br>Tatemachi<br>Hachioji,<br>Tokyo 193-09440998
201	Tokyo Metropolitan Health and Medical Treatment Corporation Ebara Hospital 4-5-10 Higashi-Yukigaya<br>Ota-ku,<br>Tokyo 145-0065

Site number	Name and Address of Committee
**Japan**	
202	Nippon Medical School Chiba Hokusoh Hospital IRB<br>1715, Kamagari, Inzaishi<br>Chiba 270-1694
203	Maebashi Red Cross Hospital IRB 3-21-36<br>Asahi Chou<br>Maebashi,<br>Gunma 371-0014
208	Yachiyo Hospital 2-2-7 Sumiyoshi-cho,<br>Anjo-city,<br>Aichi 446-8510
274	National Hospital Organization Matsumoto Medical<br>Center IRB 811, Kotobukitoyooka,<br>Matsumoto,<br>Nagano 399-0021
276	Nippon Medical School Musashi Kosugi Hospital<br>IRB 1-396, Kosugimachi, Nakaharaku,<br>kawasaki,<br>kanagawa 211-8533
277	Shinozuka Hospital IRB 105-1 Shinozuka,<br>Fujioka-city,<br>Gunma 375-0017
281	Kashiwado Hospital IRB 2-21-8 Nagazu, Chuo-ku,<br>Chiba-shi,<br>Chiba 260-86560854
**Netherlands**	
70<br>72<br>80<br>160	Vrije Universiteit Medisch Centrum Medisch<br>Ethische Toetsingscommissie<br>Postbus 7057<br>Amsterdam, NH 1007 MB

Site number	Name and Address of Committee
**New Zealand**	
54<br>107	Multi Region Ethics Committee Ministry of Health<br>PO Box 5013<br>No. 1 The Terrace,<br>Wellington 6145
**Poland**	
143<br>146<br>147<br>150	Komisja Bioetyczna przy Warszawskim<br>Uniwersytecie Medycznym w Warszawie ul. Zwirki i<br>Wigury 61,<br>Warszawa 02-091
**Portugal**	
161<br>162<br>163	Comissao de Etica Para a Investigaçao Clinica<br>Parque Da Saude De Lisboa.<br>Av. Do Brasil,<br>53 PAV. 17-A,<br>LISBOA 1749-004
**Slovakia**	
135<br>136	Eticka komisia Vseobecnej nemocnice Rimavska<br>Sobota Eticka komisia Vseobecnej nemocnice<br>Rimavska Sobota<br>Srobarova 1,<br>Rimavska Sobota 979 12
136	Eticka komisia UN Bratislava Eticka komisia UN<br>Bratislava<br>Nemocnica Ruzinov<br>Ruzinovska 6,<br>Bratislava 826 06
**South Africa**	
83<br>86	Pharma Ethics 123 Amcor Road<br>Lyttelton Manor,<br>Centurion 0157

Site number	Name and Address of Committee
**Spain**	
14	Comite Etico de Ensayos Clinicos Agencia de Ensayos Clínicos<br>Hospital Clinic de Barcelona<br>Servicio de Farmacia<br>C/ Villarroel,<br>170 Esc 6B sótano<br>08036 Barcelona
15	Comite Etico De Investigacion Clinica<br>Parc de Salut MAR Comité Ético de Investigación Clínica Parc de Salut MAR<br>Consorci Mar Parc de Salut de Barcelona<br>Parc de Recerca Biomèdica de Barcelona<br>(dcho. 163.03),<br>C/ Doctor Aiguader, 88, 1ª planta<br>08003 Barcelona
18	Hospital Mutua de Terrassa Comité Ético de Investigación Clínica<br>Hospital Mutua de Terrassa<br>Servicio de Farmacia<br>Plaça Dr. Robert, 5, Sótano -1<br>08221 Terrassa - Barcelona
19	Hospital Universitario La Paz Secretaría Técnica del Comité Ético de Investigación Clínica - Área 5<br>Hospital Universitario La Paz<br>Edificio Hospital General<br>Pº de la Castellana,<br>261 Plta. 8ª Despacho 818 - 819<br>28046 Madrid
20	Ceic De Burgos Y Soria Comité Ético de Investigación Clínica<br>Complejo Asistencial Universitario de Burgos<br>Hospital Universitario de Burgos<br>Unidad de Investigación<br>Avda. del Cid, 96<br>09005 Burgos

Site number	Name and Address of Committee
**Spain**	
23	Comité Ético de Investigación Clínica - Área 7<br>Hospital Clínico San Carlos<br>Servicio de Farmacología Clinica<br>Unidad de Coordinación de Ensayos Clínicos<br>1ª planta, Ala norte,<br>puerta G<br>C/ Prof. Martín Lagos, s/n<br>28040 Madrid
24	Comité Ético de Investigación Clínica - Área 2<br>Hospital Universitario de La Princesa<br>C/ Diego de León, 62<br>28006 Madrid
25	Comité Ético de Investigación Clínica<br>Hospital General Universitario de Elche<br>Edificio de Salud Mental 3ª planta<br>C/ Camí de L'Almazara, 11<br>03203 Elche – Alicante
26	Comité Ético de Investigación Clínica<br>Hospital Universitario Virgen de la Arrixaca<br>Planta 0 de Policlínico<br>Ctra. de Madrid - Cartagena, s/n<br>30120 El Palmar - Murcia
51	Complejo Hospitalario de Caceres Comité Ético de Investigación Clínica de Cáceres<br>Hospital Ntra. Sra. de la Montaña<br>Avda. de España, 2<br>10004 Cáceres
214	Comite Etico de Investigacion Clinica de Las Islas Baleares Comité Etico de Investigación Clinica de las Islas Baleares (CEIC-IB)<br>Consellería de Salut I Consum<br>Camino de Jesús, 38<br>07011 Palma de Mallorca - Baleares

Site number	Name and Address of Committee
**Sweden**	
4<br>215	Regionala etikprovningsnamnden i Stockholm FE 289<br>Stockholm 171 77
**Switzerland**	
127	Ethikkommission beider Basel EKBB Hebelstrasse 53<br>Basel CH-4056
**United Kingdom**	
60	NRES Committee London ¿ South East Room 4W/10, 4th Floor West,<br>Charing Cross Hospital,<br>Fulham Palace Road,<br>London, W6 8RF<br><br>Sheffield Health and Social Care NHS Research Development Unit,<br>Fulwood House,<br>Old Fulwood Road,<br>Sheffield S10 3TH<br><br>STH NHS Foundation Trust Research Department 1st Floor,<br>11 Broomfield Road,<br>Sheffield S10 2SE

Site number	Name and Address of Committee
**United Kingdom**	
61	NRES Committee London ¿ South East Room<br>4W/10, 4th Floor West,<br>Charing Cross Hospital,<br>Fulham Palace Road,<br>London, W6 8RF<br><br>Cardiff and Vale University Health Board University Hospital of Wales,<br>Commercial Clinical Trials,<br>Research & Development Office,<br>Second Floor, Tower Block 2,<br>Room 3 (2TB2 R3),<br>Heath Park,<br>Cardiff CF14 4XW
62<br>67	NRES Committee London ¿ South East Room<br>4W/10, 4th Floor West,<br>Charing Cross Hospital,<br>Fulham Palace Road,<br>London, W6 8RF
65	Avon and Wiltshire Mental Health Partnership NHS Trust Research and Development,<br>The Blackberry Centre,<br>Blackberry Hill Hospital,<br>Manor Road, Fishponds,<br>Bristol BS16 2EW<br><br>NRES Committee London,<br>South East Health Research Authority,<br>Ground Floor, Skipton House<br>80 London Road<br>London SE1 6LH

Site number	Name and Address of Committee
**United Kingdom**	
66	NHS Greater Glasgow and Community Primary Care, Community & Mental Health LREC South Glasgow & Clyde REC,<br>R&D Directorate,<br>1st Floor - The Tennent Institute,<br>Western Infirmary,<br>38 Church Street,<br>Glasgow G11 6NT<br><br>NRES Committee London ¿ South East Room 4W/10, 4th Floor West,<br>Charing Cross Hospital,<br>Fulham Palace Road,<br>London, W6 8RF
166	Imperial College London and Imperial College Healthcare NHS Trust AHSC Joint Research Office,<br>Room GM14,<br>St.Mary 's Hospital, Faculty of Medicine<br>Ground Mezzanine Floor,<br>Praed Street Wing,<br>London W2 1PG<br><br>NRES Committee London ¿ South East Room 4W/10, 4th Floor West,<br>Charing Cross Hospital,<br>Fulham Palace Road,<br>London, W6 8RF

Site number	Name and Address of Committee
**United Kingdom**	
168	Northumberland, Tyne and Wear NHS Trust<br>Research Department,<br>St Nicholas Hospital,<br>Jubilee Road,<br>Gosforth,<br>Newcastle upon Tyne NE3 3XT<br><br>NRES Committee London ¿ South East Room<br>4W/10, 4th Floor West,<br>Charing Cross Hospital,<br>Fulham Palace Road,<br>London, W6 8RF<br><br>The Newcastle upon Tyne Hospitals NHS Foundation<br>Trust Royal Victoria Infirmary,<br>Queen Victoria Road,<br>Newcastle upon Tyne NE1 4LP
175	Northampton General Hopsital NHS Trust Research<br>& Development Centre,<br>Cliftonville,<br>Northampton NN1 5BD<br><br>NRES Committee London ¿ South East Room<br>4W/10, 4th Floor West,<br>Charing Cross Hospital,<br>Fulham Palace Road,<br>London, W6 8RF

Site number	Name and Address of Committee
**United States**	
216<br>217<br>218<br>219<br>220<br>221<br>222<br>223<br>224<br>227<br>228<br>229<br>230<br>232<br>233<br>235<br>236<br>238<br>239<br>241<br>242<br>251<br>252<br>253<br>254<br>255<br>256<br>258<br>260<br>279<br>280<br>286<br>289	Western Institutional Review Board 3535 7th Ave. SW,<br>Olympia, WA 98502
240	Springfield Committee for Research Involving Human Subjects P.O. Box 19616<br>801 North Rutledge Street,<br>Springfield, IL 62702

Site number	Name and Address of Committee
**United States**	
244	Greenwich Hosptial Institutional Review Board 5 Perryridge Road,<br>Greenwich, CT 06830
245	Abington Memorial Hospital Institutional Review Board,<br>1200 Old York Road,<br>Abington, PA 19001-3788
246	Providence Health & Services Institutional Review Board,<br>Building A,<br>5251 NE Glisan Street,<br>3rd Floor,<br>Portland, OR 97213
261	Michigan State<br>University Biomedical and Health IRB,<br>207 Olds Hall,<br>East Lansing, MI 48824
263	Thomas Jefferson University Office of Scientific Affairs,<br>Division of Human Subjects Protection<br>Suite 1100<br>1015 Chestnut Street,<br>Philadelphia, PA 19107
264	Medical University of South Carolina Office of Research Integrity,<br>19 Hagood Avenue, Suite 601<br>MSC857<br>Charleston, SC 29425
269	Western Institutional Review Board<br>MSC857
