# Supplementary material for: Long-term safety and tolerability of bapineuzumab in patients with Alzheimer’s disease in two phase 3 extension studies
Source: Alzheimers Res Ther. 2016 Jun 23;8:24. doi: 10.1186/s13195-016-0193-y (PMC4918115; doi:10.1186/s13195-016-0193-y)
Supplement: Additional file 2: — is a list of independent ethics committees or institutional review boards that approved the ApoE ε4 noncarrier studies. (PDF 124 kb) [file 13195_2016_193_MOESM2_ESM.pdf]

16.1.3 List of Independent Ethics Committees or Institutional Review Boards

A list of the following is included:

- The List of Independent Ethics Committees, sorted by country and site, for sites that screened subjects.

Independent Ethics Committees

Site Number	Name and Address of Committee
**Australia**	
53	Austin Health Human Research Ethics Committee<br>145 Studley Road<br>Heidelberg, Vic 3084
55	Hollywood Private Hospital Research Ethics Committee<br>Monash Avenue,<br>Nedlands, WA 6009
56	Northern Sydney / Central Coast Area Health Service HREC<br>Human Research Ethics Committee, Royal North Shore Hospital<br>Pacific Hwy<br>St Leonards, NSW 2065
57	Central Northern Adelaide Health Service Ethics of Human Research Committee (TQEH & LMH)<br>The Queen Elizabeth Hospital<br>28 Woodville Road<br>WOODVILLE SOUTH, SA 5011
58	Royal Adelaide Hospital Research Ethics Committee<br>Royal Adelaide Hospital<br>Level 3, Hanson Centre<br>North Terrace<br>Adelaide, SA 5000
**Belgium**	
47<br>48<br>49	Commissie Medische Ethiek van de Universitaire Ziekenhuizen KU<br>Campus Gasthuisberg E330<br>Herestraat 49<br>Leuven 3000
**Chile**	
126<br>176	Comite Etico Cientifico, Servicio de Salud Metropolitano Oriente<br>Av. Salvador 364<br>Providencia Santiago, RM 7500922

Site Number	Name and Address of Committee
**Finland**	
3<br>6	Pohjois-Savon sairaanhoitopiirin ky<br>Tutkimuseettinen toimikunta<br>Rakennus 10 (4. krs)<br>PL 1777Kuopio 70211
**France**	
109<br>110<br>111<br>115<br>117<br>119<br>120<br>121<br>123<br>124<br>157<br>158<br>291	CPP Ile de France 3<br>Hôpital TARNIER<br>89, rue dAssas<br>PARIS 75006
**Italy**	
29	Comitato Etico Indipendente<br>dell'IRCCS Fondazione S. Lucia di Roma<br>Via Ardeatina, 306, Roma 00179
33	Comitato Etico<br>della Fondazione IRCCS Istituto Neurologico Carlo Besta di Milano<br>Via Celoria, 11<br>Milano 20133

Site Number	Name and Address of Committee
**Japan**	
177	National Hospital Organization Kokura Medical Center<br>10-1 Harugaoka Kokuraminami-ku<br>Kitakyusyu-shiFukuoka 802-8533
178	Yokohama City University Medical Center IRB<br>4-57 Urafunecho Minami-kuYokohama, Kanagawa 232-0024
179	National Hospital Organization Minami-Okayama Medical Center IRB<br>4066 Hayashima, Hayashima-cho, Tsukubo-gun<br>Okayama 701-0304
180	Juntendo University Hospital IRB<br>3-1-3 HongoBunkyo-ku, Tokyo 113-8431
182	National Hospital Organization Chiba-East Hospital<br>673 Nitona-chou Chuou-kuChiba, Chiba 260-8712
183	Kagawa University Hospital IRB<br>1750-1 Ikenobe Miki-choKita-gun, Kagawa 761-0793
184	National Hospital Organization Shizuoka Institute of Epilepsy and Neurological Disorders<br>886 Urushiyama<br>Aoi-kuShizuoka, Shizuoka 420-8688
185	Osaka City University Hospital<br>1-5-7, Asahi-machi,<br>Abeno-ku,Osaka, Osaka, 545-8586
186	Juntendo Tokyo Koto Geriatric Medical Center IRB<br>3-3-20, Shinsuna<br>Koto-ku, Tokyo 136-0075
187	Tokyo Medical University Hospital<br>6-7-1 Nishi-ShinjukuShinjuku-ku, Tokyo 160-0023
189	Okayama University Hospital IRB<br>2-5-1 Shikata-cho, Kita-ku Okayama, Okayama 700-8558

Site Number	Name and Address of Committee
**Japan**	
190	National Hospital Organization Minami-Kyoto Hospital IRB<br>11 Ashihara, Naka,Jouyou-shi, Kyoto 610-0113
191	National Hospital Organization Hiroshima-nishi Medical Center<br>4-1-1 KubaOtake city, Hiroshima 739-0696
192	National Hospital Organization Tokyo National Hospital IRB<br>3-1-1 Takeoka, Kiyose-shi, Tokyo 204-8585
193	National Hospital Organization Maizuru Medical Center IRB<br>2410 Aza Yukinaga, Maizuru, Kyoto 625-8502
194	Kansai Medical University Takii Hospital<br>10-15 Fujizono-cho Moriguchi, Osaka 570-8507
196	Kobe University Hospital IRB<br>7-5-2 kusunoki-cho, Chuo-kuKobe, Hyogo 650-0017
197	Nagoya City University Hospital IRB<br>1 Kawasumi Mizuho-cho, Mizuho-ku,<br>Nagoya, Aichi, 467-8602
198	Osaka University Hospital IRB<br>Institutional Review Board<br>2-15 YamadaokaSuita, Osaka 565-0871
200	Tokyo Medical University Hachioji Medical Center<br>1163 TatemachiHachioji, Tokyo 193-0944
201	Tokyo Metropolitan Health and Medical Treatment Corporation Ebara Hospital<br>4-5-10 Higashi-YukigayaOta-ku, Tokyo 145-0065
208	Yachiyo Hospital<br>2-2-7 Sumiyoshi-cho, Anjo-city, Aichi 446-8510
274	National Hospital Organization Matsumoto Medical Center IRB<br>811, Kotobukitoyooka, Matsumoto, Nagano 399-0021
276	Nippon Medical School Musashi Kosugi Hospital IRB<br>1-396, Kosugimachi, Nakaharaku,<br>kawasaki, kanagawa 211-8533

Site Number	Name and Address of Committee
**Japan**	
308	Himorogi Psychiatric Institute IRB<br>1-20-10 SugamoToshima-ku, Tokyo 170-0002
**Netherlands**	
72<br>80<br>160	Vrije Universiteit Medisch Centrum<br>Medisch Ethische Toetsingscommissie<br>Postbus 7057<br>Amsterdam, NH 1007 MB
**New Zealand**	
107	Multi Region Ethics Committee<br>Ministry of Health<br>PO Box 5013<br>Wellington
**Poland**	
143<br>146<br>147<br>150<br>282	Komisja Bioetyczna przy Warszawskim Uniwersytecie<br>Medycznym w Warszawie<br>ul. Zwirki i Wigury 61<br>Warszawa 02-091
**Portugal**	
161<br>162<br>163	Comissao de Etica Para a Investigaçao Clinica<br>PARQUE DA SAUDE DE LISBOA.<br>AV. DO BRASIL, 53 PAV. 17-A<br>LISBOA, LISBOA 1749-004
**Slovakia**	
135	Eticka komisia Vseobecnej nemocnice Rimavska Sobota<br>Srobarova 1<br>Rimavska Sobota 979 12

Site Number	Name and Address of Committee
**Slovakia**	
136	Eticka komisia Vseobecnej nemocnice Rimavska Sobota<br>Srobarova 1<br>Rimavska Sobota 979 12<br>Slovakia<br><br>Eticka komisia UN Bratislava<br>Nemocnica Ruzinov<br>Ruzinovska 6<br>Bratislava 826 06
**South Africa**	
86<br>89	Pharma Ethics<br>123 Amcor Road<br>Lyttelton ManorCenturion 0157
**Spain**	
14	Hospital Clinic i Provincial de Barcelona<br>Comite Etico de Investigacion Clinica<br>Villarroel, 170<br>Barcelona, Barcelona 08036<br><br>COMITE ETICO DE INVESTIGACION CLINICA Parc de Salut MAR, COMITE ETICO DE INVESTIGACION CLINICA, EDIFICIO PRBB<br>C/ DOCTOR AIGUADER, Nº 88<br>BARCELONA, BARCELONA 08003

Site Number	Name and Address of Committee
**Spain**	
15	COMITE ETICO DE INVESTIGACION CLINICA Parc de Salut MAR<br>COMITE ETICO DE INVESTIGACION CLINICA<br>EDIFICIO PRBB<br>C/ DOCTOR AIGUADER, Nº 88<br>BARCELONA, BARCELONA 08003<br><br>CEIC Parc de Salut Mar<br>Comite Etico de Investigacion Clinica<br>Secretaria Tecnica. Parc de Recerca Biomedica de Barcelona (Dcho. 163.03)<br>C/ Doctor Aiguader, 88<br>Barcelona, Barcelona 08003
16	Hospital de la Santa Creu i Sant Pau<br>COMITE ETICO DE INVESTIGACION CLINICA<br>AVDA. SANT ANTONI MARIA CLARET, 167<br>BARCELONA, BARCELONA 08025<br><br>COMITE ETICO DE INVESTIGACION CLINICA Parc de Salut MAR<br>COMITE ETICO DE INVESTIGACION CLINICA<br>EDIFICIO PRBB<br>C/ DOCTOR AIGUADER, Nº 88<br>BARCELONA, BARCELONA 08003
18	Hospital Mutua de Terrassa<br>Comite Etico de Investigacion Clinica<br>Pl. Dr. Robert, 5<br>Terrassa, Barcelona 08221<br><br>COMITE ETICO DE INVESTIGACION CLINICA Parc de Salut MAR<br>COMITE ETICO DE INVESTIGACION CLINICA<br>EDIFICIO PRBB<br>C/ DOCTOR AIGUADER, Nº 88<br>BARCELONA, BARCELONA 08003

Site Number	Name and Address of Committee
**Spain**	
19	Hospital Universitario La Paz<br>Comite Etico de Investigacion Clinica<br>Hospital General Planta 8ª<br>Paseo de la Castellana, 261<br>Madrid, Madrid 28046<br><br>COMITE ETICO DE INVESTIGACION CLINICA Parc de Salut MAR<br>COMITE ETICO DE INVESTIGACION CLINICA<br>EDIFICIO PRBB<br>C/ DOCTOR AIGUADER, Nº 88<br>BARCELONA, BARCELONA 08003
20	COMITE ETICO DE INVESTIGACION CLINICA Parc de Salut MAR<br>COMITE ETICO DE INVESTIGACION CLINICA<br>EDIFICIO PRBB, C/ DOCTOR AIGUADER, Nº 88<br>BARCELONA, BARCELONA 08003, CEIC DE BURGOS Y SORIA, Hospital General Yague<br>Avda del Cid, 96BURGOS 09005
21	Hospital 12 de Octubre<br>Instituto de Investigacion Hospital 12 de Octubre (i+12)<br>Area de Gestion de Proyectos - Unidad Administrativa CEIC<br>Centro de Actividades Ambulatorias, Bloque D - Planta 6ª<br>Avda de Cordoba s/nMadrid, Madrid 28041<br><br>COMITE ETICO DE INVESTIGACION CLINICA Parc de Salut MAR<br>COMITE ETICO DE INVESTIGACION CLINICA<br>EDIFICIO PRBB<br>C/ DOCTOR AIGUADER, Nº 88<br>BARCELONA, BARCELONA 08003

Site Number	Name and Address of Committee
**Spain**	
23	Hospital Clinico San Carlos<br>Comite Etico de Investigacion Clinica<br>Ciudad Universitaria. Planta 1ª - Ala Norte - Puerta G<br>C/ Doctor Martin Lagos s/n<br>Madrid, Madrid 28040<br><br>COMITE ETICO DE INVESTIGACION CLINICA Parc de Salut MAR<br>COMITE ETICO DE INVESTIGACION CLINICA<br>EDIFICIO PRBB<br>C/ DOCTOR AIGUADER, Nº 88<br>BARCELONA, BARCELONA 08003
24	Hospital Universitario de la Princesa<br>Comite Etico de Investigacion Clinica<br>C/ Diego de Leon 62<br>Madrid 28006<br><br>COMITE ETICO DE INVESTIGACION CLINICA Parc de Salut MAR<br>COMITE ETICO DE INVESTIGACION CLINICA<br>EDIFICIO PRBB<br>C/ DOCTOR AIGUADER, Nº 88<br>BARCELONA, BARCELONA 08003

Site Number	Name and Address of Committee
**Spain**	
25	Hospital General de Elche<br>Comite Etico de Investigacion Clinica<br>Cami de l'Almazara, 11<br>Elche, Alicante 03203<br><br>COMITE ETICO DE INVESTIGACION CLINICA Parc de Salut MAR<br>COMITE ETICO DE INVESTIGACION CLINICA<br>EDIFICIO PRBB<br>C/ DOCTOR AIGUADER, Nº 88<br>BARCELONA, BARCELONA 08003
51	Complejo Hospitalario de Caceres<br>Comite Etico de Investigacion Clinica<br>Avda. Pablo Naranjo, s/n<br>Caceres, Caceres 10003<br><br>COMITE ETICO DE INVESTIGACION CLINICA Parc de Salut MAR<br>COMITE ETICO DE INVESTIGACION CLINICA<br>EDIFICIO PRBB<br>C/ DOCTOR AIGUADER, Nº 88<br>BARCELONA, BARCELONA 08003

Site Number	Name and Address of Committee
**Spain**	
212	Hospital Universitario Ramon y Cajal<br>Comite Etico de Investigacion Clinica<br>Secretaria del Comite - Planta 2ª Dcha.<br>Ctra. de Colmenar Viejo, Km. 9.100<br>Madrid, Madrid 28034<br><br>COMITE ETICO DE INVESTIGACION CLINICA Parc de Salut MAR<br>COMITE ETICO DE INVESTIGACION CLINICA<br>EDIFICIO PRBB<br>C/ DOCTOR AIGUADER, Nº 88<br>BARCELONA, BARCELONA 08003
214	COMITE ETICO DE INVESTIGACION CLINICA Parc de Salut MAR<br>COMITE ETICO DE INVESTIGACION CLINICA<br>EDIFICIO PRBB<br>C/ DOCTOR AIGUADER, Nº 88<br>BARCELONA, BARCELONA 08003<br><br>Comite Etico de Investigacion Clinica de Las Islas Baleares<br>Conselleria de Salut i Consum<br>Camino de Jesus, 38<br>Palma de Mallorca, Islas Baleares 07011
**Sweden**	
004	Regionala etikprovningsnamnden i Stockholm<br>FE 289<br>Stockholm 171 77
**Switzerland**	
127	Ethikkommission beider Basel EKBB<br>Hebelstrasse 53 Basel CH-4056

Site Number	Name and Address of Committee
**United Kingdom**	
60	NRES Committee London-South East<br>South East Coast Strategic Health Authority<br>Preston Hall<br>Aylesford, Kent ME20 7NJ<br><br>Sheffield Health and Social Care NHS<br>Research Development Unit<br>Fulwood House<br>Old Fulwood Road<br>Sheffield S10 3TH<br><br>NRES Committee London ¿ South East<br>Room 4W/10, 4th Floor West<br>Charing Cross Hospital<br>Fulham Palace Road<br>London, UK W6 8RF<br><br>STH NHS Foundation Trust Research Department<br>1st Floor<br>11 Broomfield Road<br>Sheffield S10 2SE
61	NRES Committee London South East<br>Room 4W/10, 4th Floor West<br>Charing Cross Hospital<br>Fulham Palace Road<br>London, UK W6 8RF<br><br>NRES Committee London-South East<br>South East Coast Strategic Health Authority<br>Preston Hall<br>Aylesford, Kent ME20 7NJ<br><br>Cardiff and Vale University Health Board<br>University Hospital of Wales<br>Commercial Clinical Trials, Research & Development Office<br>Second Floor, Tower Block 2, Room 3 (2TB2 R3)<br>Heath ParkCardiff CF14 4XW

Site Number	Name and Address of Committee
**United Kingdom**	
62	NRES Committee London ¿ South East<br>Room 4W/10, 4th Floor West<br>Charing Cross Hospital, Fulham Palace Road<br>London, UK W6 8RF<br><br>NRES Committee London-South East<br>South East Coast Strategic Health Authority<br>Preston Hall<br>Aylesford, Kent ME20 7NJ<br><br>Research & Development Directorate, Brighton & Sussex University Hospitals NHS Trust, Clinical Investigaton & Research Unit, Royal Sussex County Hospital, Level 5<br>Thomas Kemp Tower<br>Eastern RoadBrighton BN25BE
65	NRES Committee London South East<br>Room 4W/10, 4th Floor West<br>Charing Cross Hospital<br>Fulham Palace Road<br>London, UK W6 8RF<br><br>NRES Committee London-South East<br>South East Coast Strategic Health Authority<br>Preston Hall<br>Aylesford, Kent ME20 7NJ<br><br>NRES Committee London South East<br>Health Research Authority<br>Ground Floor, Skipton House<br>80 London Road<br>London SE1 6LH<br><br>Avon and Wiltshire Mental Health Partnership NHS Trust<br>Research and Development<br>The Blackberry Centre<br>Blackberry Hill Hospital<br>Manor Road, Fishponds Bristol BS16 2EW

Site Number	Name and Address of Committee
**United Kingdom**	
66	NRES Committee London ¿ South East<br>Room 4W/10, 4th Floor West<br>Charing Cross Hospital<br>Fulham Palace Road<br>London, UK W6 8RF<br><br>NRES Committee London-South East<br>South East Coast Strategic Health Authority<br>Preston Hall<br>Aylesford, Kent ME20 7NJ<br><br>NHS Greater Glasgow and Community Primary Care,<br>Community & Mental Health LREC<br>South Glasgow & Clyde REC<br>R&D Directorate, 1st Floor - The Tennent Institute<br>Western Infirmary<br>38 Church Street Glasgow G11 6NT
159	NRES Committee London South East<br>Room 4W/10, 4th Floor West<br>Charing Cross Hospital<br>Fulham Palace Road<br>London, UK W6 8RF<br><br>NRES Committee London-South East<br>South East Coast Strategic Health Authority<br>Preston Hall<br>Aylesford, Kent ME20 7NJ<br><br>Kings Health Partners<br>Joint Clinical Trials Office<br>Floor 16, Tower Wing<br>Guy's Hospital<br>Great Maze Pond London SE1 9RT

Site Number	Name and Address of Committee
**United Kingdom**	
166	NRES Committee London South East<br>Room 4W/10, 4th Floor West, Charing Cross Hospital<br>Fulham Palace Road, London, UK W6 8RF<br>NRES Committee London-South East<br>South East Coast Strategic Health Authority, Preston Hall,<br>Aylesford, Kent ME20 7NJ<br><br>Imperial College London and Imperial College Healthcare<br>NHS Trust, AHSC Joint Research Office, Room GM14<br>St. Mary 's Hospital, Faculty of Medicine<br>Ground Mezzanine Floor<br>Praed Street Wing London W2 1PG
168	The Newcastle upon Tyne Hospitals NHS Foundation Trust<br>Royal Victoria Infirmary<br>Queen Victoria Road<br>Newcastle upon Tyne NE1 4LP<br>NRES Committee London ¿ South East<br>Room 4W/10, 4th Floor West<br>Charing Cross Hospital<br>Fulham Palace Road<br>London, UK W6 8RF<br><br>Northumberland, Tyne and Wear NHS Trust<br>Research Department<br>St Nicholas Hospital<br>Jubilee Road<br>GosforthNewcastle upon Tyne NE3 3XT<br><br>NRES Committee London-South East<br>South East Coast Strategic Health Authority<br>Preston Hall<br>Aylesford, Kent ME20 7NJ
